# SG-APSIC1055: Hand hygiene challenges among the ancillary team during the COVID-19 pandemic

**DOI:** 10.1017/ash.2023.45

**Published:** 2023-03-16

**Authors:** Qinnan Liu, Kamini Devi, Ismail Bin Sazali, Tan Kwee Yuen, Shaiful Bahri Maroni, King Richard Jay Ganotisi, Quek Bak Siang, Ling Moi Lin

**Affiliations:** Singapore General Hospital, Singapore; D/O Magesparan, Singapore General Hospital, Singapore; Singapore General Hospital, Singapore; Singapore General Hospital, Singapore; Singapore General Hospital, Singapore; Singapore General Hospital, Singapore; Singapore General Hospital, Singapore; Singapore General Hospital, Singapore

## Abstract

**Objectives:** Ancillary staff members perform operational support functions and play an active role in enhancing the patient care experience. Infection prevention practices among ancillary staff play a critical role in preventing transmission of microorganisms, which ensures the safety of patients. Low hand hygiene compliance was found among porters in a cross-institutional hand hygiene audit in 2021. A quality improvement team was formed to improve hand hygiene compliance, especially during the COVID-19 pandemic. **Methods:** A focus-group discussion and survey were conducted to understand hand hygiene knowledge and challenges among porters. Using the findings, the team initiated Glo–germ education tools, pocket alcohol hand-rub agents, pocket moisturizer, poster display, and a toolbox messaging system via conversion of group roll call to satellite-area roll call. Respective satellite teams were sent hand hygiene reminders, and prompt corrective action was taken following noncompliance events. Analytic comparisons of pre- and postsurvey data were performed using the χ^2^ test, and *P* < .05 was regarded as statistically significant. **Results:** In total, 572 ancillary staff participated in the survey. Knowledge of hand hygiene practices improved significantly following the interventions, as shown in the comparison of pre- and postintervention results: knowledge of the hand hygiene steps (*P* < .001), knowledge of the duration of hand rub (*P* < .001), and knowledge of duration of handwashing (*P* < .001). Also, 295 staff members (97.68%) stated that implementation measures increased their awareness of the importance of hand hygiene. Moreover, the hand hygiene compliance rate improved from 77.8% to 100%. There were no significant differences related to sex (*P* = .089), age group (*P* = .355), years of working (*P* = .359), education level (*P* = .268), or difficulty in reading English (*P* = .906). **Conclusions:** Evaluating staff hand hygiene knowledge and understanding the challenges faced among porters helped toward the development of appropriate interventions and assurance of success in project.

